# Red Grape Skin Polyphenols Blunt Matrix Metalloproteinase-2 and -9 Activity and Expression in Cell Models of Vascular Inflammation: Protective Role in Degenerative and Inflammatory Diseases

**DOI:** 10.3390/molecules21091147

**Published:** 2016-08-29

**Authors:** Nadia Calabriso, Marika Massaro, Egeria Scoditti, Mariangela Pellegrino, Ilaria Ingrosso, Giovanna Giovinazzo, Maria Annunziata Carluccio

**Affiliations:** 1 National Research Council–Institute of Clinical Physiology (CNR-IFC), Laboratory of Nutrigenomic and Vascular Biology, Lecce 73100, Italy; nadia.calabriso@ifc.cnr.it (N.C.); marika.massaro@ifc.cnr.it (M.M.); egeria.scoditti@ifc.cnr.it (E.S.); mariangela_pellegrino@yahoo.it (M.P.); 2 Department of Biological and Environmental Science and Technologies (DISTEBA), University of Salento, Lecce 73100, Italy; 3 National Research Council–Institute of Science of Food Production, Lecce 73100, Italy; ilariaingrosso1@virgilio.it (I.I.); giovanna.giovinazzo@ispa.cnr.it (G.G.)

**Keywords:** antioxidants, gelatinases, endothelium, monocytes, grape skin polyphenolic extracts, gene expression, inflammation, stilbenes, flavonols, phenolic acids

## Abstract

Matrix metalloproteinases (MMPs) are endopeptidases responsible for the hydrolysis of various components of extracellular matrix. MMPs, namely gelatinases MMP-2 and MMP-9, contribute to the progression of chronic and degenerative diseases. Since gelatinases’ activity and expression are regulated by oxidative stress, we sought to evaluate whether supplementation with polyphenol-rich red grape skin extracts modulated the matrix-degrading capacity in cell models of vascular inflammation. Human endothelial and monocytic cells were incubated with increasing concentrations (0.5–25 μg/mL) of Negroamaro and Primitivo red grape skin polyphenolic extracts (NSPE and PSPE, respectively) or their specific components (0.5–25 μmol/L), before stimulation with inflammatory challenge. NSPE and PSPE inhibited, in a concentration-dependent manner, endothelial invasion as well as the MMP-9 and MMP-2 release in stimulated endothelial cells, and MMP-9 production in inflamed monocytes, without affecting tissue inhibitor of metalloproteinases (TIMP)-1 and TIMP-2. The matrix degrading inhibitory capacity was the same for both NSPE and PSPE, despite their different polyphenolic profiles. Among the main polyphenols of grape skin extracts, *trans*-resveratrol, *trans*-piceid, kaempferol and quercetin exhibited the most significant inhibitory effects on matrix-degrading enzyme activities. Our findings appreciate the grape skins as rich source of polyphenols able to prevent the dysregulation of vascular remodelling affecting degenerative and inflammatory diseases.

## 1. Introduction

Matrix metalloproteinases (MMPs) are a family of zinc-containing enzymes with proteolytic activity against the extracellular matrix [1]. As such MMP expression and activity play an important role in physiological processes associated with homeostasis regulation, host defence and tissue repair [1]. Although usually tightly controlled, events of MMP activity deregulation occur in several pathological processes involving matrix remodelling such as in cancer invasion and metastasis [2] and atherosclerotic plaque rupture and erosion [3]. Based on substrate preference, we can divided MMPs into four categories: collagenases, gelatinases, stromelysins and membrane-type MMPs.

Among MMPs, gelatinase-A (MMP-2) and gelatinase-B (MMP-9) are recognized as the key enzymes in the degradation of type IV collagen, the major component of basement membrane and are emerging as critically involved in chronic inflammatory and degenerative diseases [4,5]. Notably, several lines of evidence show a positive correlation between MMP-2 and -9 expression and tumour progression [6] and metastasis in various human cancers [7,8,9].

Furthermore, MMP-2 and -9 activities are also increased in the acute coronary syndrome [10] and during the formation, progression and instability of atherosclerotic lesions [11,12]. Finally, various studies point to an association between increased levels of gelatinases in patients with neurodegenerative diseases [4,13,14]. Under these pathophysiological conditions, relevant sources of MMP-2 and -9, are represented by endothelial cells lining the vessel wall and monocytes/macrophages [9,15]. Both monocytes/macrophages and endothelial cells secrete gelatinases in response to inflammatory stimuli and oxidative stress, and, in turn, exacerbated release of reactive oxygen species (ROS) have been shown to regulate MMP gene expression and activation [16,17]. Correspondingly, the ROS scavenger *N*-acetylcysteine has been shown to inhibit both gelatinolytic activity and MMP-9 expression in experimental atheroma [17]. Therefore, gelatinases represent an attractive therapeutic target susceptible to modulation by antioxidants, and there is a growing interest in identifying natural compounds able to inhibit MMPs activity and matrix degradation.

It is now largely recognized that a daily consumption of fruits and vegetables may actively preserve human health and reduce the risk of chronic and degenerative diseases, through mechanisms including the regulation of matrix remodelling by dietetic polyphenols [18,19,20]. In particular, since the enunciation of the “French paradox” [21], much attention has been focused on the health properties of polyphenols from red wine [22,23,24]. Experimental studies showed that red wine polyphenolic compounds strongly inhibited MMP-2 expression and activation in vascular smooth muscle cells and reduced endothelial migration [25,26]. Concordantly, we have recently shown that red wine polyphenolic extracts exhibited multiple anti-inflammatory and anti-atherosclerotic properties [27], and specific red wine polyphenols, including *trans*-resveratrol and quercetin, inhibited endothelial migration and MMP-9 activity and expression [28]. However, experimental and clinical studies showed that the amount of red wine necessary to provide a level of phenolic compounds adequate to obtain significant healthful effects is too high to avoid the deleterious alcohol consequences [29,30]. Recently grape berries skins, as by-products of winemaking processes, have been revaluated as rich source of polyphenols [31], but studies on the characterization of extracts composition and biological activities are poor.

On this background, we characterized the berry skin polyphenolic composition of two red grape cultivars, Negroamaro and Primitivo, typical of South Italy (Apulia, Italy). We studied the effects of skin polyphenolic extracts on extracellular matrix degradation, in vascular wall and circulating cellular models of inflammation, by analysing cell invasion capacity and MMP-9 and MMP-2 gelatinolytic activity and expression as well as tissue inhibitor of metalloproteinases (TIMP)-1 and TIMP-2 expression. Finally, we investigated, in both endothelial cells and monocytes, the effects of the most represented red grape skin polyphenols, on the expression and activity of MMP-9 and MMP-2 under pro-inflammatory conditions.

## 2. Results

### 2.1. Polyphenolic Profile and Antioxidant Property of Negroamaro and Primitivo Grape Skin Extracts

In the present study we determined polyphenolic composition and related antioxidant properties of Negroamaro and Primitivo skin polyphenolic extracts (henceforth NSPE and PSPE, respectively). We reported that the total polyphenols amount in the two grape skin extracts was not significantly different (Table 1). Notably, phenolic acids were the most representative polyphenols of both extracts, followed by flavonols and stilbenes (Table 1). However, in NSPE and PSPE differences were detected in the amount of specific polyphenols (Table 1). Even though total flavonol levels were unchanged, kaempferol and quercetin-3-*O*-rutinoside were significantly higher in PSPE than in NSPE, while quercetin and quercetin-3-*O*-glucoside were more abundant in NSPE. Regarding stilbenes, the ratio between *trans*-resveratrol and its glycosylated form *trans*-piceid changed significantly in the two grape varieties, being *trans*-resveratrol higher in PSPE and *trans*-piceid higher in NSPE. Although two extracts did not significantly differ in the total amount of phenolic acids, gallic acid was present mainly in PSPE.

We also evaluated the free radical scavenging capacities of NSPE and PSPE by using the trolox equivalent antioxidant capacity (TEAC) assay, which can measure antioxidant capacities of hydrophilic and lipophilic compounds in the same sample [32]. We found that both total and water-soluble fraction antioxidant capacity did not significantly change between NSPE and PSPE. However, the TEAC values of the fat-soluble fraction significantly varied from 1.80 ± 0.01 to 6.32 ± 0.04 μmol·Trolox/g for NSPE and PSPE, respectively; this difference could be due to the presence of higher amount of phenolic aglycon compounds, including *trans*-resveratrol and kaempferol (Table 2).

### 2.2. Red Grape Skin Polyphenol Extracts Prevent MMP-9 and MMP-2 Gelatinolytic Activity and Cell Invasion in Inflamed Endothelium

**Figure 1:**
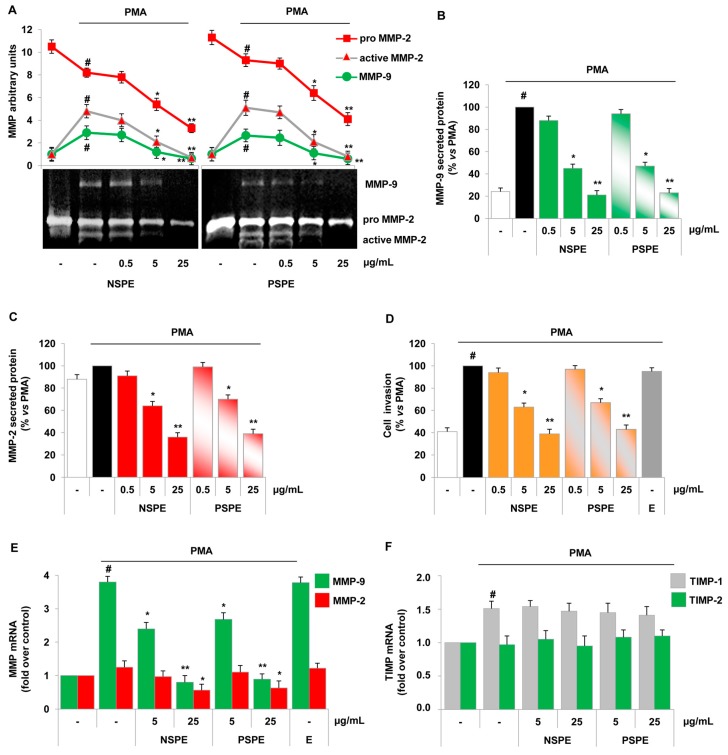
Inhibitory effects of red grape skin polyphenol extracts on MMP-2 and MMP-9 activity and expression and endothelial invasion. HUVEC were incubated with NSPE and PSPE (0.5, 5, 25 µg/mL) or ethanol vehicle (E, 0.025% *v*/*v*) for 1 h and then stimulated with 20 nmol/L PMA, after which culture media were collected and analysed by gelatin zymography (**A**) or by ELISA (**B**,**C**); invasiveness was performed by transwell cell invasion assay (**D**); mRNA levels of MMP-9, MMP-2 and TIMP-1, TIMP-2 were assessed by qRT-PCR (**E**,**F**). Results are expressed as mean ± SD and are representative of four separate experiments yielding similar results. # *p* < 0.01 vs. control. * *p* < 0.05 and ** *p* < 0.01 vs. PMA alone.

We investigated the impact of NSPE and PSPE on endothelial invasiveness evaluating the ability of the extracts to modulate the gelatinolytic capacity, the extracellular matrix degradation and the cell invasion under pro-inflammatory conditions mimicked by treatment with phorbol myristate acetate (PMA). PMA is a tumour promoter and recognized pro-angiogenic factor, and it activates various signalling cascades including protein kinase C. We used PMA as an agent able to induce migration and invasion of endothelial cells through the production of pro-inflammatory markers including gelatinases. To study the effect of red grape skin polyphenol extracts on endothelial release of gelatinases, we exposed human vein endothelial cells (HUVEC) to increasing concentration of NSPE and PSPE (0.5, 5, 25 µg/mL) for 1 h before cell stimulation with PMA for further 24 h. Conditioned media were then collected and the release and activity of gelatinases were investigated. Figure 1A shows that endothelial cells constitutively released an immature form of MMP-2 (proMMP-2, 72 kDa), however under PMA challenge, they expressed both pro and active MMP-2 forms (68 and 62 kDa) as well as MMP-9 (92 kDa), as assessed by gelatin zymography (Figure 1A). When we exposed HUVEC to NSPE and PSPE before stimulation, the gelatinolytic activities of MMP-2 and MMP-9 were significantly reduced in a concentration-dependent manner with a comparable potency for both extracts (Figure 1A).

Both NSPE and PSPE reduced PMA-induced MMP-9 production by about 60% at 5 µg/mL, and lowered it at the control levels at 25 µg/mL. Moreover, both extracts at 5 µg/mL reduced the stimulated production of active MMP-2 and proMMP-2 by about 58% and 33%, respectively (Figure 1A). Active MMP-2 release was nullified at 25 µg/mL of NSPE and PSPE, thus resetting the active MMP-2/proMMP-2 ratio to the basal unstimulated condition. In agreement with anti-gelatinolytic activities, we showed that NSPE and PSPE inhibited in a concentration-dependent manner, both MMP-9 and MMP-2 protein release, at ELISA, with 5 µg/mL the lower effective inhibitory concentration (Figure 1B,C).

Since gelatinase activity is a key factor involved in matrix degradation, endothelial cell migration and invasiveness, we investigated the effects of red grape skin polyphenol extracts on PMA-induced endothelial cell invasion as a functional counterpart of the release and activity of gelatinases. Consistent with their effect on gelatinolytic capacity, NSPE and PSPE pre-treatment, but not the corresponding vehicle ethanol, reduced the endothelial cell invasion by about 35% at 5 µg/mL and brought it back to unstimulated control levels at 25 µg/mL (Figure 1D).

Being 5 and 25 µg/mL the effective concentrations of red grape skin polyphenol extracts, we have chosen these doses for all subsequent assays. The gelatinolytic activity is controlled at multiple levels including the regulation of MMPs and TIMPs gene expression. With the aim to deeper understand the mechanisms of action of red grape skin polyphenolic extracts, we explored their potential abilities to interfere with mechanisms of gene expression regulation directly measuring the mRNA levels of MMP-9, MMP-2 and related TIMP-1 and TIMP-2 by qRT-PCR. Our results showed that PMA significantly increased the MMP-9 mRNA levels by about 3.8-fold and TIMP-1 by about 1.5-fold, while it was devoid of any significant effect on MMP-2 and TIMP-2 mRNA expression (Figure 1E,F).

In accordance with zymography results, NSPE and PSPE treatment suppressed, in a concentration-dependent manner, the MMP-9 mRNA levels with a significant inhibitory effect at 5 µg/mL, while at the higher concentration (25 µg/mL) they also shrank MMP-2 mRNA level in endothelial cells (Figure 1E). TIMP-1 and TIMP-2 mRNA expression levels were not modified by NSPE or PSPE (Figure 1F). Of note, the inhibitory effects of red grape skin polyphenol extracts occurred only under pro-inflammatory conditions and in the absence of toxic effects, as determined by the MTT assay, protein content and cellular counts (data not shown). Taken together, our data suggested that NSPE and PSPE attenuated the invasion of endothelial cells through the down-regulation of MMP-2 and MMP-9 expression, release and activity.

### 2.3. Red Grape Skin Polyphenol Extracts Suppress MMP-9 Release and Expression in Inflammatory Monocytes

**Figure 2:**
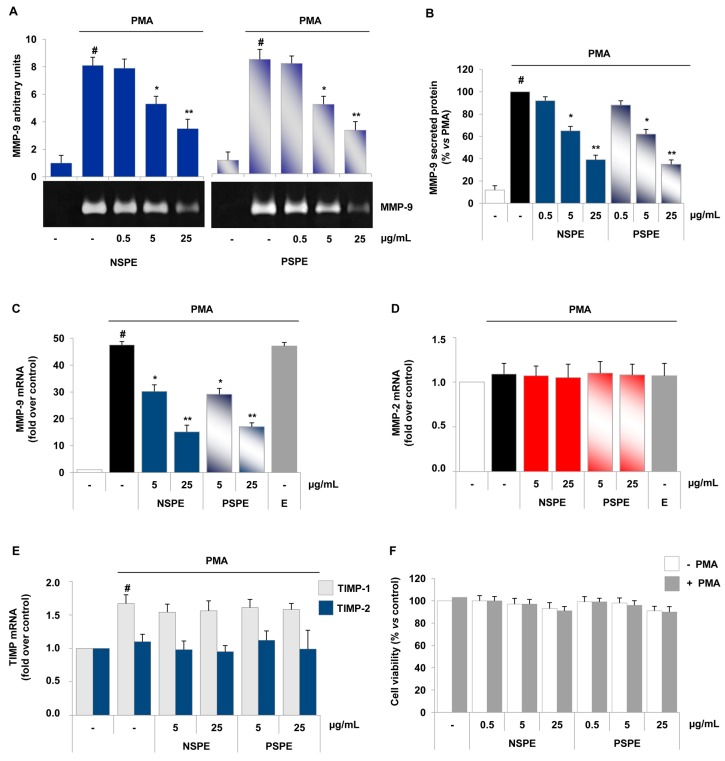
Inhibitory effects of red grape skin polyphenol extracts on MMP-9 activity and expression in inflamed monocytes. U937 were incubated with NSPE and PSPE (0.5, 5, 25 µg/mL) or ethanol vehicle (E, 0.025% *v*/*v*) for 1 h and then stimulated with 20 nmol/L PMA. Media were collected and analysed by gelatin zymography (**A**) or ELISA (**B**); mRNA levels of MMP-9, MMP-2, TIMP-1 and TIMP-2 were assessed by qRT-PCR (**C**–**E**); cell viability was determined by the MTT assay (**F**). Results expressed as mean ± SD are representative of four separate experiments yielding similar results. # *p* < 0.01 vs. control. * *p* < 0.05 and ** *p* < 0.01 vs. PMA alone.

U937 monocytes under inflamed conditions, mimicked by PMA treatment, expressed and released high levels of MMP-9 (Figure 2). At gelatin zymography, red grape skin polyphenol extracts attenuated the PMA-induced secretion of MMP-9 by U937 cells in a concentration-dependent manner (Figure 2A,B). Similarly to endothelial cells, 5 µg/mL was the lowest effective concentration of NSPE and PSPE able to reduce, by about 40%, the release of MMP-9 in inflamed monocytes. In accordance with MMP-9 release, levels of MMP-9 mRNA in PMA-treated cells were notably lowered by NSPE and PSPE as evidenced by qRT-PCR (Figure 2C). Noteworthily, in our experimental conditions, we found that PMA induced mainly MMP-9 in U937 monocytoid cells, whereas MMP-2 was not detectable at gelatin zymography or present at very low concentrations at qRT-PCR. MMP-2 mRNA remained not changed after PMA stimulation and/or treatment of NSPE and PSPE (Figure 2D). Moreover, PMA only induced TIMP-1 mRNA expression, but NSPE and PSPE did not significantly modify either TIMP-1 or TIMP-2 (Figure 2E). Cell treatment with NSPE or PSPE ranging from 0.5 to 25 µg/mL did not show significant decrease in viability of U937 cells at the MTT assay (Figure 2F), indicating that NSPE and PSPE inhibitory effects on MMP-9 gelatinolytic activity and expression was not due to cytotoxicity.

### 2.4. Specific Red Grape Skin Polyphenols Differently Modulate Gelatinase Activity and Expression in Inflamed Endothelial Cells and Monocytes

**Figure 3:**
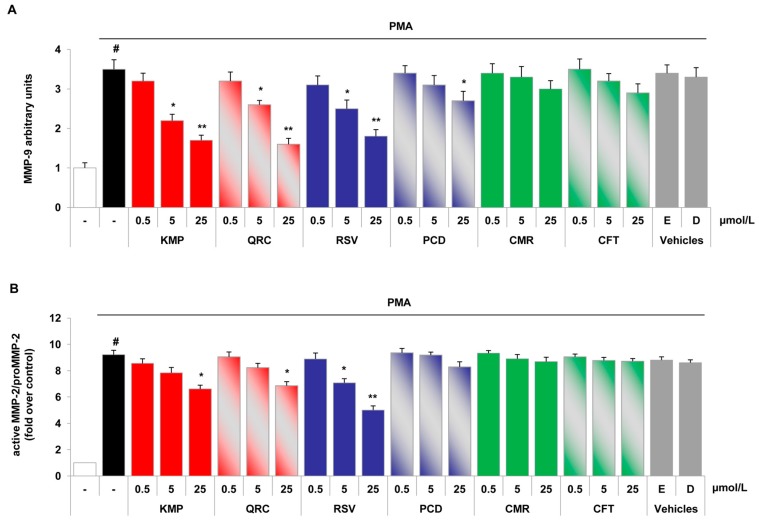
Differential effects of pure polyphenols on the stimulated release of gelatinases in endothelial cells. HUVEC were pre-treated with increasing concentrations of each polyphenol (0.5, 5 and 25 μmol/L) or vehicle (ethanol, E, 0.025% *v*/*v*; DMSO, D, 0.025% *v*/*v*) for 1 h and then stimulated with 20 nmol/L PMA, after which culture media were collected and analysed by gelatin zymography. Bar graphs show the quantification of MMP-9 levels (**A**) and MMP-2/pro-MMP-2 levels (**B**) of four independent experiments (mean ± SD). # *p* < 0.01 vs. control. * *p* < 0.05 and ** *p* < 0.01 vs. PMA alone.

**Figure 4:**
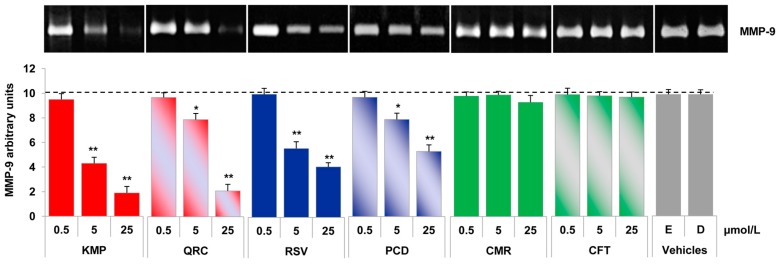
Differential effects of pure polyphenols on the stimulated release of MMP-9 in monocytes. U937 were pre-treated with increasing concentrations of each polyphenol (0.5, 5 and 25 μmol/L) or vehicle (ethanol, E, 0.025% *v*/*v*; DMSO, D, 0.025% *v*/*v*) for 1 h and then stimulated with 20 nmol/L PMA, after which culture media were collected and analysed by gelatin zymography. Bar graphs show the quantification of MMP-9 levels of four independent experiments (mean ± SD). PMA release specified by dashed line. * *p* < 0.05 and ** *p* < 0.01 vs. PMA alone.

To dissect the specific contribution of polyphenols present in NSPE and PSPE (Table 1 and Table 3), we investigated the effects of representative polyphenols of the different polyphenol classes including stilbenes, flavonols and phenolic acids on gelatinase activity and expression. To this purpose, HUVEC and U937 cells were treated with chemically synthesized polyphenols including stilbenes, flavonols and soluble acids at increasing concentrations (0.5, 5, 25 μmol/L) before PMA stimulation, and conditioned media were analysed by gelatin zymography (Figure 3 and Figure 4).

As shown in Figure 3A, in endothelial cells *trans*-resveratrol, kaempferol and quercetin significantly inhibited in a concentration-dependent manner MMP-9 production with a significant inhibition at 5 μmol/L. *Trans*-piceid exhibited inhibitory effect on MMP-9 release only at the highest concentrations (25 μmol/L), whereas soluble acids (*p*-coumaric and caftaric acids) were devoid of any significant effects in our experimental conditions (Figure 3A). In addition to MMP-9, the tested polyphenols also affected the release of active and proMMP-2 in inflamed endothelial cells. Figure 3B shows that *trans*-resveratrol at 5 and 25 μmol/L significantly reduced, in a dose-dependent manner, the active MMP-2/proMMP-2 ratio. Tested flavonols reduced significantly active MMP-2/proMMP-2 levels only at 25 μmol/L, while *p*-coumaric and caftaric acids as well as *trans*-piceid did not significantly modify MMP-2 levels.

Similarly to endothelial cells, the PMA-stimulated MMP-9 release was prevented by pure polyphenols in monocytes (Figure 4). In particular, stilbenes as well as kaempferol and quercetin reduced MMP-9 release in a concentration-dependent manner, with 5 μmol/L as the lowest effective concentration. *p*-coumaric and caftaric acids were devoid of any significant effects.

## 3. Discussion

There is growing interest in the utilization of red grape skins as a source of polyphenols, and subsequent exploitation of their nutraceutical and healthy properties [33]. However, red grape skins derived from different cultivars may have distinguishable polyphenolic composition and possibly diverse biological efficacy. Difference in the extent of beneficial effects of grape was linked with the grape cultivars and growing area impact on the total polyphenol composition [34,35].

In the present study, we characterized the polyphenolic extracts obtained from the skins of two varieties of red grapes, Negroamaro and Primitivo, growing in the Apulia area (Southern Italy). We demonstrated the ability of polyphenolic extracts, NSPE and PSPE, to inhibit cell invasiveness and the related matrix-degradation by inflamed endothelial cells and monocytes, pointing to beneficial effect on the vascular system. Specifically, NSPE and PSPE decreased in a concentration-dependent manner the endothelial release and activity of MMP-9 as well as the MMP-2 production and activation in response to PMA with a significant effect already evident at concentrations starting from 5 μg/mL. Besides endothelial cells, NSPE and PSPE prevented the stimulated release of MMP-9 also in inflamed monocytes/macrophages, again at concentrations starting from 5 μg/mL. We have previously revealed that PMA induced endothelial migration through involvement of MMP, and that MMP inhibitors abolished PMA-induced endothelial migration [28]. Since, in the present study we showed that NSPE and PSPE exhibited anti-MMP activity, we hypothesize that these two extracts inhibited the endothelial invasiveness by interfering with the extracellular matrix degradation and subsequent endothelial migration.

To the best of our knowledge, this is the first report investigating the inhibitory effects of grape skin polyphenols on the MMP-9 and MMP-2 release and gelatinolytic activities in human monocytes and endothelial cells under inflammatory conditions. Our results extend previous studies showing vascular protective effects of grape skin polyphenolic extracts such as inhibition of thrombosis [36], angiogenesis [37], and endothelial dysfunction [38]. The present findings are consistent with previous clinical study highlighting that in healthy human volunteers the daily moderate consumption of wine rich in polyphenols improved vascular function accompanied by an acute decrease in the plasma MMP-9 concentration and a significant decrease in plasma levels of oxidizing species. These beneficial biological effects were paralleled by red wine polyphenol content, as revealed by urinary phenolic metabolites excretion [39]. Moreover, in LDL receptor knockout mice, red wine was also able to reduce atherosclerosis lesions by inhibiting the production and activation of MMP-2 [40]. Red wine polyphenolic extracts effectively reduced also the development of colon carcinoma by blunting tumour vascularization and inhibiting pro-angiogenetic key factors including MMP-2 [41]. Finally, polyphenolic extracts from grape pomace were shown to prevent the up-regulation of colonic MMP-9 expression, recognized as potentially responsible for the accelerated extracellular matrix breakdown and remodelling in inflammatory bowel disease, thus providing prevention against colon inflammation [42].

The activity of MMP can be regulated by endogenous TIMP, which play an important role in the physiological maintenance of the extracellular matrix and the pathogenesis of vascular disease [43]. Notably, among different TIMPs, TIMP-1 has known to regulate preferentially MMP-9 activity, and TIMP-2 to control MMP-2 activity. The quantitative ratio between MMPs and TIMPs expression finally determines the activities of the proteases [44]. We showed that both extracts reduced PMA-induced MMP-9 and MMP-2 expression, without affect TIMP-1 and -2, thus lowering gelatinases/TIMPs ratio and consequent MMP-9 and MMP-2 gelatinolytic activity.

In order to investigate the mechanisms underlying the inhibition of gelatinase activity by NSPE and PSPE, we analysed their effects on the expression of TIMP-1 and TIMP-2. In both endothelial cells and monocytes, PMA induced TIMP-1 but not TIMP-2 mRNA, but the pre-conditioning with grape skin polyphenolic extracts did not affect TIMP-1 or TIMP-2 mRNA levels.

Gene expression of MMPs can be also regulated by mechanisms involving the activation of several transcription factors by pro-inflammatory stimuli. The promoter regions of MMP-9 and MMP-2 genes show remarkable conservation of regulatory elements, including potential binding sites for the redox sensitive transcription factors nuclear factor-kappa B (NF-κB) and activator protein-1 (AP-1) [45,46,47]. We have previously shown that PMA induced MMP-9 expression through the activation of NF-κB [48], which was inhibited by pure red wine polyphenols [28]. Moreover, our recent findings demonstrated that red wine polyphenolic extracts from Negroamaro and Primitivo inhibited the activation of NF-κB and AP-1 [27], this suggesting that red grape skin polyphenolic extracts could inhibit MMP-9 and MMP-2 interfering with their gene expression regulatory mechanisms. Accordingly, we found that NSPE and PSPE reduced the PMA-induced mRNA levels of MMP-9 in both endothelial cells and monocytes in a concentration-dependent manner. However, the inhibitory effects on MMP-2 mRNA levels occurred only at high concentration of NSPE and PSPE in endothelial cells, while were absent in U937 monocytes. Overall, we found that grape skin polyphenolic extracts were able to down-regulate the expression of gelatinases but not TIMP-1 and TIMP-2, so reducing the MMPs/TIMPs ratio and consequently MMP-9 and MMP-2 activity, as well as matrix-degrading capacity and invasiveness. Noteworthy, we revealed that both NSPE and PSPE similarly contributed to the reduction of gelatinases expression and activity as well as the extracellular matrix degrading capacities, although the different polyphenolic profile and lipophilic antioxidant capacity of the two extracts, suggesting a compensatory and/or synergistic effects of different polyphenolic compounds. However, the total antioxidant capacity of red grape skin polyphenolic extracts was not significantly different and was related to anti-MMP activity, suggesting that the total (hydrophilic and lipophilic) radical scavenger properties of NSPE and PSPE, measured by TEAC assay, could explained their inhibitory effects on gelatinase expression and activity which are known to be modulated by ROS.

In order to dissect the specific contribution of isolate polyphenols in NSPE and PSPE, we investigated the effects of flavonoids, namely flavonols (kaempferol and quercetin) as well as non-flavonoids such as stilbenes (*trans*-resveratrol and *trans*-piceid) and soluble acids (*p*-coumaric acid and caftaric acid) on stimulated MMP-9 and MMP-2 release in endothelial cells and monocytes. We found that both flavonols and stilbenes significantly reduced the stimulated production of gelatinases in endothelial cells as well as in monocytes in a concentration dependent manner. The most potent MMP-9 inhibitors were *trans*-resveratrol, kaempferol and quercetin being effective already at 5 μmol/L; whereas *trans*-piceid was more effective in monocytes compared with endothelial cells. We have also highlighted that *trans*-resveratrol reduced the ratio of active MMP-2 and proMMP-2 with a significant inhibition already at 5 μmol/L. Kaempferol and quercetin decreased the active MMP-2/proMMP-2 ratio only at high concentrations (25 μmol/L). Finally, *p*-coumaric and caftaric acids were devoid of any significant inhibitory effect on gelatinolytic activity in endothelial cells as well as in monocytes. The differential gelatinolytic efficacy of pure polyphenols can provide the explanation of the similar gelatinolytic activity of NPSE and PSPE. Indeed, NPSE presented higher levels of quercetin and *trans*-piceid than PSPE, which in turn presented higher levels of *trans*-resveratrol and kaempferol. The present data are consistent with previous studies in different in vitro cellular models, including our previous findings, showing the inhibitory gelatinolytic effects of individual red wine polyphenols [28,49,50,51,52,53,54]. However, to our knowledge, the present data are the first demonstration of the MMP-9 inhibitory role of *trans*-piceid and kaempferol both in stimulated endothelial cells and monocytes as well as the inhibitory effect on MMP-2 release by kaempferol, quercetin and *trans*-resveratrol.

However, a limitation of the present study was that our findings derived from in vitro models, therefore they cannot be directly extrapolated to humans. Moreover, the health effects of polyphenols are largely dependent on their bioavailability and metabolism and synergy with other dietary factors. Polyphenol bioavailability appears to differ deeply, due to polyphenol structure, glycosylation and/or conjugation levels with other compounds [55]. Metabolism of polyphenol also depends on the characteristics of the compounds. The aglycones and some glucosides can be absorbed in the small intestine, meanwhile other forms must be hydrolyzed before they can be absorbed [56]. However, even though the forms of polyphenols appearing in the circulation after absorption could be different from those found in red grape skin used in this study, free polyphenols (i.e., their aglycones) can be locally generated in vivo from their metabolites at sites of inflammation, for example by the action of glucuronidases and sulfatases [57]. As a possible relevance of our findings, we found that the lowest effective concentration of polyphenolic extracts is similar to that can be attained in vivo through diets enriched with plant products containing complex mixtures of various polyphenols [55]. In details, the protective effects of NSPE and PSPE were detected at concentrations as low as 5 µg/mL, in which the concentration of flavonols (kaempferol, quercetin) was inferior to 1 µmol/L and *trans*-resveratrol and *trans*-piceid were inferior to 0.15 and 1 µmol/L, respectively. Several studies showed that the plasma concentrations of total metabolites, resulting from digestive and hepatic activity after ingestion of a single dose of polyphenols, ranged from 0 to 4 μmol/L with an intake of 50 mg aglycone equivalents, and the relative urinary excretion ranged from 0.3% to 43% of the ingested dose, depending on the polyphenol [55].

There are numerous studies demonstrating the bioavailability of the main bioactive polyphenols in grape and wine [58]. While all flavonols and stilbenes are bioavailable, different studies have drawn attention to whether wine and grape delivers sufficient amounts of these compounds to have health benefits. Nevertheless, one of the goals of functional food development is to increase levels of health-promoting compounds so that the functional food is capable of delivering an efficacious amount. Resveratrol is rapidly and extensively metabolized [58] to sulfate and glucuronide conjugates. Predictions of concentrations of resveratrol in systemic circulation vary [59], for example about 2.4 nmol/L unmodified resveratrol and 180 nmol/L total resveratrol from a dose equivalent to two glasses of red wine, and about 9 μmol/L authentic resveratrol and about 680 μmol/L total resveratrol from a high, but pharmacologically relevant, dose of resveratrol of 100 mg per kg body weight [60]. In the same study, the authors considered the effect of food on resveratrol absorption by co-administering the resveratrol with a standard high-fat meal. They found that while the rate of absorption decreased with food, the extent of absorption was unaffected. The bioavailability of quercetin has received more attention than that of resveratrol possibly as consequence of the fact that quercetin has been identified as the most abundant polyphenol in the diet [61]. Studies reported a median maximum plasma concentration of quercetin of 431 nmol/L from a dose of 150 mg quercetin [62]. Quercetin naturally occurs as glycoside forms that are either hydrolyzed by intestinal enzymes or by the colonic microflora before they can be absorbed [63]. Quercetin is of particular interest, because micromolar plasma concentration can be achievable through diet and the elimination of quercetin metabolites is quite slow, this favoring accumulation in plasma with repeated intakes. Quercetin is thus more bioavailable than resveratrol. Anyway, in a recent study humans consuming small daily doses of red grape powder, i.e., in an amount equivalent to only 3.5–7 mg of resveratrol, showed an high rate of resveratrol absorption and considerable presence time in the circulation [64]. One of the factors that may improve resveratrol solubility and hence its bioavailability is the glycosylation of the resveratrol parent compound. Therefore, since NSPE and PSPE contained stilbenes mainly as piceid, the glycoside form of *trans*-resveratrol, it could be expected that they could be effective in vivo also at low doses. Finally, the observed inhibitory effects of pure polyphenols, both flavonols and stilbenes, occurred at concentrations higher than those that can be found in red grape skin extracts (Table 3), this suggesting the occurrence of a synergism among different polyphenols in the extracts and that low doses of extracts could exhibit, also in vivo, synergic bioactive health-promoting effects.

Overall, we here demonstrated that polyphenols extracted from red grape skins exhibited beneficial effects in the context of pathological tissue remodelling by inhibiting the expression and activity of gelatinases in endothelial cells and monocytes under inflamed conditions and that the inhibitory effect of red grape skin polyphenols on MMP-2 and -9 activity and expression observed in the present study occurs at concentrations that are likely to be achieved in the plasma of subjects after moderate red grape skin consumption.

## 4. Experimental Section

### 4.1. Materials

Samples of autochthonous red grape variety (*Vitis vinifera* L.), Primitivo and Negramaro, were produced in Salento (southern area of the Apulia Region, Italy). *trans*-Resveratrol and *trans*-piceid were obtained from ICN Biomedicals (South Chillicothe Road, Aurora, OH, USA), quercetin-3-*O*-glucoside, myricetin and kaempferol from ExtraSynthese (Genay, France), quercetin, *p*-coumaric acid and caftaric acid from Sigma (St. Louis, MO, USA) as well as all other reagents when not otherwise specified. The materials for cell cultures were obtained from Gibco/BRL (Gaithersburg, MD, USA).

### 4.2. Preparation of Polyphenolic Extracts

To assess a representative sample of the vineyard, grape sampling was carried out by taking into account the variability of the positions of the fruit on the cluster, the cluster on the vine, and the vine in the vineyard as well as the sun exposures. Healthy grape berries were snipped from the clusters, the skin from 50 healthy berries, randomly collected, were manually separated from pulp and seeds, weighed and then frozen in liquid nitrogen, grinded with a blender until fine powder and lyophilized and stored at 5 °C until analysis. The samples (100 mg of dry weight) were resuspended with 2 mL of water and extracted three times with methanol 99% and methyl *tert*-butyl ether (2:1:1 *v*/*v*/*v*). Cell debris was removed by centrifugation (4000 rpm) for 5 min, the supernatant evaporated to dryness, under reduced pressure, at 40 °C and re-dissolved in 70% ethanol (500 µL). Samples were protected from light during the analysis process.

### 4.3. Identification and Quantification of Phenolic Compounds

Different compounds present in grape skin extracts were separated by RP-HPLC DAD (Agilent 1100 HPLC system (Agilent Technologies Inc., Santa Clara, CA, USA). The separation was performed on a C_18_ column (5 UltraSphere, 80 A pore, 25 mm), with a linear gradient from 20% to 60% acetonitrile, in 55 min (solvent A: 1% H_3_PO_4_, solvent B: 100% acetonitrile) with a flow of 1 mL/min at 25 °C. The chromatographic analysis performed with UV detectors was based on the comparison of results with retention time of external standards. The metabolite concentrations were obtained by deduction from the calibration curves and were expressed in µg/g. Recovery was determined by adding known amounts of different metabolites and values were between 85%–93%. The absence of total anthocyanins pigments in skin extracts and alcoholic solutions were measured at 520 nm after appropriate dilution (1 N HCl, pH 1.0) (0.02–1 absorbance unit) [58].

### 4.4. Antioxidant Activity of Grape Skin Polyphenolic Extracts

Antioxidant activity of grape skin extracts was carried out according to D’introno et al. [65] with minor modifications. Grape skins were frozen in liquid nitrogen and ground with a blender to a fine powder. Dried grape skin powder (100 mg) was extracted with 50 mM sodium phosphate buffer (2 mL, pH 7.5) and ethyl acetate (5 mL). The homogenate was centrifuged at 4000 g for 10 min in order to separate the aqueous from the organic phase. The hydrophilic and lipophilic antioxidant capabilities were measured in the two phases, collected separately. The capability of the aqueous and organic phases to scavenge the 2,2′-azinobis-(3-ethylbenzothiazoline-6-sulphonate) (ABTS) radical cations was compared with a standard dose-response curve obtained using 6-hydroxy-2,5,7,8-tetramethylchroman-2-carboxylic acid (Trolox), and was expressed as Trolox equivalents (TE) of dry weight (µmol TE/g d.w.). The combined hydrophilic and lipophilic antioxidant capabilities were considered as the total antioxidant activity.

### 4.5. Cell Culture and Treatment

Human umbilical vein endothelial cells (HUVEC) were harvested, characterized and grown in M199 medium containing 10% foetal bovine serum (FBS) as described [66]. Cells were obtained from discarded umbilical vein and treated anonymously conforming with principles outlined in the Declaration of Helsinki. The human monocytic cell line U937 was purchased from the American Type Culture Collection (Rockville, MD, USA) and grown in RPMI medium 1640 containing 10% FBS. U937 cells were maintained at density less than 1 × 10^6^ cells/mL to prevent cell differentiation.

Before treatment, red grape skin dry powders were dissolved in 70% ethanol and resulting extracts, NSPE or PSPE, were used at the same concentration after an appropriate dilution in culture medium. For treatment, confluent endothelial cells or U937 cells were shifted to medium supplemented with 2.5% FBS or, for zymography, to serum-free medium containing 0.1% human serum albumin for 4 h and subsequently incubated in the absence or presence of increasing concentrations of NSPE or PSPE (0.5, 5, 25 μg/mL) or pure polyphenols (0.5, 5, 25 μmol/L) for 1 h, and then triggered with phorbol 12-myristate 13-acetate (PMA) (20 nmol/L) for additional 4–24 h. Stock solutions of polyphenols (100 mmol/L) included *trans*-resveratrol, *p*-coumaric and caftaric acid in absolute ethanol, *trans*-piceid in 70% ethanol, quercetin and kaempferol in DMSO. As vehicle control, HUVEC were incubated with appropriate amount of each solvent (<0.025% *v*/*v*). These concentrations of ethanol or DMSO had no effect on any of the parameters measured in this study. Cellular toxicity by treatments was checked through a variety of techniques including cell count, morphology, protein content and MTT (3-(4, 5-dimethylthiazolyl-2)-2,5-diphenyltetrazolium bromide) assays.

### 4.6. Gelatinase Activity

The activity of gelatinase A (MMP-2) and gelatinase B (MMP-9) in conditioned media was evaluated by gelatin zymography. Conditioned media were centrifuged at 800× *g*, mixed with non-reducing Laemmli sample buffer, and subjected to electrophoresis on 1% sodium dodecyl sulfate (SDS)/7.5% polyacrylamide gel containing 1 mg/mL gelatin. After electrophoresis, SDS was removed from the gel by washings with 2.5% Triton X-100, and then incubated in a reaction buffer (50 mmol/L TRIS-HCl, pH 7.4, 10 mmol/L CaCl_2_ and 0.05% polyoxyethylene lauryl ether) overnight at 37 °C. At the end of incubations, gels were stained with Coomassie Brilliant Blue. Clear bands against the blue background indicate the presence of proteinolytic activity. To confirm the identity of MMPs, similar gels were incubated in the reaction buffer containing either 20 mmol/L EDTA, an inhibitor of MMPs, or 1 mmol/L phenylmethanesulfonyl fluoride (PMSF), a serine proteinase inhibitor. The addition of PMSF did not alter MMPs activity, whereas treatment with EDTA completely abolished it.

### 4.7. MMP-9 and MMP-2 Protein Release

The levels of secreted MMP-9 and MMP-2 protein in conditioned media were quantified using the highly specific Biotrack ELISA system (GE Healthcare, Freiburg, Germany), according to manufacturer’s instructions.

### 4.8. Cell Invasion Assay

Cell invasion was determined using transwell chambers (Cell Biolabs, Inc. San Diego, CA, USA) with 6.5 mm polycarbonate filters of 8 μm pore size. Each filter was pre-coated with 100 μL of 1:20 (*v*:*v*) diluted Matrigel in cold medium to form a thin continuous film on the upper chamber. Endothelial cells (5 × 10^4^/400 μL) were suspended in serum-free medium and placed on the upper chamber and then incubated with NSPE or PSPE (0.5, 5, 25 μg/mL). The lower chambers were added culture medium containing 10% FBS and 20 nmol/L PMA. After 16 h incubation, endothelial cells on the upper chamber were completely wiped away using cotton swabs. Endothelial cells on the lower chamber were fixed with methanol for 10 min, stained with crystal violet, and then counted under a microscope.

### 4.9. Quantitative Reverse Transcription-Polymerase Chain Reaction Analysis

HUVEC or U937 cells were treated with NSPE or PSPE for 1 h before stimulation with 20 nmol/L PMA for 4 h. Total RNA was isolated by using the TRIzol reagent (Invitrogen, Carlsbad, CA, USA) according to the manufacturer’s protocol. For quantitative polymerase chain reaction, total RNA (2 μg) was converted into first-strand cDNA by using the High Capacity cDNA Reverse Transcription Kit (Applied Biosystems, Monza, Italy). The quantitative RT-PCR (qRT-PCR) was performed in the Bio-Rad Biosystems CFX384 Touch Real-Time PCR Detection System, by using SYBR Green PCR Master Mix (Bio-Rad, Hercules, CA, USA). The human MMP-9, MMP-2, TIMP-1, TIMP-2, cDNA fragments were amplified using primers synthesized by Sigma Genosys and reported in Table 4. The relative quantities of target gene mRNA against an internal controls, GAPDH and 18S rRNA, were measured by following a ΔCt method. The difference (ΔCt) between the main value in the triplicate samples of target gene and those of GAPDH/18S rRNA were calculate using the CFX Manager (Version 3.0) Software (Bio-Rad) and the relative quantified value was expressed as 2^−ΔCt^. Results are expressed as fold increase relative to unstimulated control (=1).

### 4.10. Statistical Analysis

Values are expressed as mean ± SD of at least four independent experiments. Differences between two groups were determined by unpaired Student’s t test. Multiple comparisons were performed by one way analysis of variance (ANOVA), and individual differences then tested by the Fisher’s protected least-significant difference test after the demonstration of significant inter-group differences by ANOVA.

## 5. Conclusions

Our findings indicate new biological properties of grape polyphenolic extracts and promote red grape skin as an exploitable source of bioactive compounds and as a potential nutraceutical for the prevention of degenerative inflammatory diseases. Moreover, the present in vitro study suggests potential mechanisms by which red grape polyphenols modulate extracellular matrix degradation and contribute to explain their protective properties in diseases where the role of vascular remodelling is crucial.

## Figures and Tables

**Table 1:** Polyphenols content (±standard deviation) of berry skin of Negroamaro and Primitivo grapes (*n* = 5). * The percent values followed by an asterisk were statistically different between NSPE and PSPE (*p* ≤ 0.05).

Polyphenols	NSPE		PSPE	
	µg/g Dry Weight	(%)	µg/g Dry Weight	(%)
**Flavonols**		**21.0**		17.5 *
Kaempferol	25.9 ± 0.4	0.3	209.7 ± 1.1	2.5 *
Kaempferol-3-*O*-glucoside	209.8 ± 1.5	2.9	180.2 ± 1.3	2.2
Quercetin	150.2 ± 1.5	2.1	118.8 ± 1.2	1.4 *
Quercetin-3-*O*-glucoside	908.8 ± 5.5	12.5	650.5 ± 4.1	7.8 *
Quercetin-3-*O*-rutinoside	180.8 ± 1.4	2.5	248.7 ± 1.5	3.0 *
Myricetin-3-*O*-glucoside	48.8 ± 0.5	0.7	54.1 ± 0.4	0.6
**Stilbenes**		**7.8**		5.2 *
*Trans*-Resveratrol	30.6 ± 0.4	0.4	54.3 ± 0.9	0.6 *
*Trans*-Piceid	536.8 ± 2.2	7.4	378.2 ± 2.5	4.5 *
**Phenolic acids**		**71.2**		**77.3**
*p*-Coumaric acid	2488.8 ± 5.5	32.2	2989.8 ± 5.5	35.9
Caftaric acid	1200.2 ± 2.9	16.5	998.8 ± 7.8	12.0
Gallic acid	1487.9 ± 2.8	20.5	2447.8 ± 3.5	29.4 *
**Total**	**7268.6**		**8327.4**	

**Table 2:** Lipophilic and hydrophilic antioxidant activity (LAA and HAA, respectively), as well as total antioxidant activity of NSPE and PSPE (*n* = 5) were expressed as Trolox equivalent (TE). * Values followed by an asterisk were statistically different between NSPE and PSPE (*p* ≤ 0.05).

	LAA	HAA	Total
	µmoles TE/g Dry Weight		
**NSPE**	1.80 ± 0.01	17.65 ± 0.23	19.45
**PSPE**	6.32 ± 0.04 *	16.47 ± 0.52	22.79

**Table 3:** Chemical structures of specific polyphenols identified in red grape skin extracts and relative concentrations in NSPE and PSPE at 5 and 25 μmol/L.

				NSPE (µg/mL)		PSPE (µg/mL)	
				5	25	5	25
STRUCTURE	POLYPHENOLS	R_1_	R_2_	µmol/L			
**FLAVONOLS**							
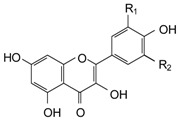	Kaempferol (KMP)	H	H	0.05	0.26	0.44	2.18
	Quercetin (QRC)	OH	H	0.35	1.74	0.23	1.16
**STILBENES**							
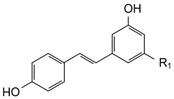	*trans*-Resveratrol (RSV)	OH	-	0.09	0.44	0.13	0.66
	*trans*-Piceid (PCD)	OGlucose	-	0.95	4.74	0.58	2.88
**SOLUBLE ACIDS**							
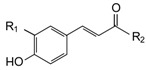	*p*-Coumaric acid (CMR)	H	OH	9.81	49.04	10.93	54.67
	Caftaric acid (CFT)	OH	C_4_O_6_H_5_	2.64	13.22	1.92	9.61

**Table 4:** Primers sequences of qRT-PCR.

Gene Name	Accession Number	Forward Primer	Reverse Primer	Size (bp)
MMP-9	NM_004994.2	5′-AAAGCCTATTTCTGCCAGGAC-3′	5′-GTGGGGATTTACATGGCACT-3′	157
MMP-2	NM_004530.4	5′-CACTTTCCTGGGCAACAAAT-3′	5′-TGATGTCATCCTGGGACAGA-3′	257
TIMP-1	NM_003254.2	5′-TGACATCCGGTTCGTCTACA-3′	5′-CTGCAGTTTTCCAGCAATGA-3′	103
TIMP-2	NM_003255.4	5′-CCAAGCAGGAGTTTCTCGAC-3′	5′-TTTCCAGGAAGGGATGTCAG-3′	121
GAPDH	NM_002046.3	5′-ATCACTGCCACCCAGAAGAC-3′	5′-TTCTAGACGGCAGGTCAGGT-3′	210
18 rRNA	NR_003286.2	5′-AAACGGCTACCACATCCAAG-3′	5′-CCTCCAATGGATCCTCGTTA-3′	155
